# Telecom-band lasing in single InP/InAs heterostructure nanowires at room temperature

**DOI:** 10.1126/sciadv.aat8896

**Published:** 2019-02-22

**Authors:** Guoqiang Zhang, Masato Takiguchi, Kouta Tateno, Takehiko Tawara, Masaya Notomi, Hideki Gotoh

**Affiliations:** 1 NTT Basic Research Laboratories, NTT Corporation, 3-1 Morinosato-Wakamiya, Atsugi, Kanagawa 243-0198, Japan.; 2 NTT Nanophotonics Center, NTT Corporation, 3-1 Morinosato-Wakamiya, Atsugi, Kanagawa 243-0198, Japan.

## Abstract

Telecom-band single nanowire lasers made by the bottom-up vapor-liquid-solid approach, which is technologically important in optical fiber communication systems, still remain challenging. Here, we report telecom-band single nanowire lasers operating at room temperature based on multi-quantum-disk InP/InAs heterostructure nanowires. Transmission electron microscopy studies show that highly uniform multi-quantum-disk InP/InAs structure is grown in InP nanowires by self-catalyzed vapor-liquid-solid mode using indium particle catalysts. Optical excitation of individual nanowires yielded lasing in telecom band operating at room temperature. We show the tunability of laser wavelength range in telecom band by modulating the thickness of single InAs quantum disks through quantum confinement along the axial direction. The demonstration of telecom-band single nanowire lasers operating at room temperature is a major step forward in providing practical integrable coherent light sources for optoelectronics and data communication.

## Teaser

We demonstrate optically pumped telecom-band lasing in single InAs/InP nanowires at room temperature.

## INTRODUCTION

The discovery and continued development of the laser have revolutionized both science and industry (*1*–*3*). The advent of miniaturized semiconductor lasers has continued with a new focus on nanowire lasers because of their great potential in the field of integrated optoelectronics (*3*–*10*). The telecom-band wavelength range (1.2 to 1.6 μm) is technologically important in optical fiber communication systems. Despite significant progress of nanowire lasers working in ultraviolet, visible, and near-infrared (<1 μm) range (*11*–*22*), there are few reports about nanowire lasers in the telecom-band wavelength range (*22*–*25*). This is partially because (i) population inversion is more difficult for long-wavelength range due to enlarged band imbalance of low-bandgap materials, and (ii) optical gain coefficient decreases with longer working wavelength.

On the one hand, much effort has been made to make telecom-band nanowire lasers by combining nanowires with external high-Q cavities, e.g., photonic crystal. Danang *et al.* (*25*) embedded InAsP nanowires into the groove of photonic crystal cavity and realized lasing at telecom band at cryogenic temperature (*22*). Kim *et al.* (*23*) also used the photonic crystal cavity effect resulting from one-dimension nanowire array and realized lasing in O and E bands at room temperature. Nevertheless, these hybrid lasers need external cavities and thus enlarge the device footprint and volume. On the other hand, from the point view of miniaturization, the single nanowire laser is much more advantageous because of its small footprint and low energy consumption. For realization of nanowire lasers in near-infrared range, InP and GaAs nanowires have been mostly used, and there have been a number of reports about GaAs and InP nanowire lasers in the wavelength range of 0.8 to 1.0 μm (*17*, *18*, *26*–*29*). For the extension of wavelength to telecom-band range, InGaAs and GaAsSb material has been used as the active layer (*19*, *30*–*32*). However, the wavelength of these nanowire lasers is still shorter than 1.3 μm (O band). Chin *et al.* (*15*) have reported near-infrared nanowire lasers with the lasing wavelength of 1.5 μm by using GaSb semiconductor material, but the nanowire laser works at cryogenic temperature. The use of only the GaSb semiconductor also makes it difficult to tune the wavelength in a relatively wide range. Up to now, it still remains chalenging to realize single nanowire lasers that cover the full telecom-band (O, E, C, and L bands) range and operate at room temperature.

**Fig. 1:**
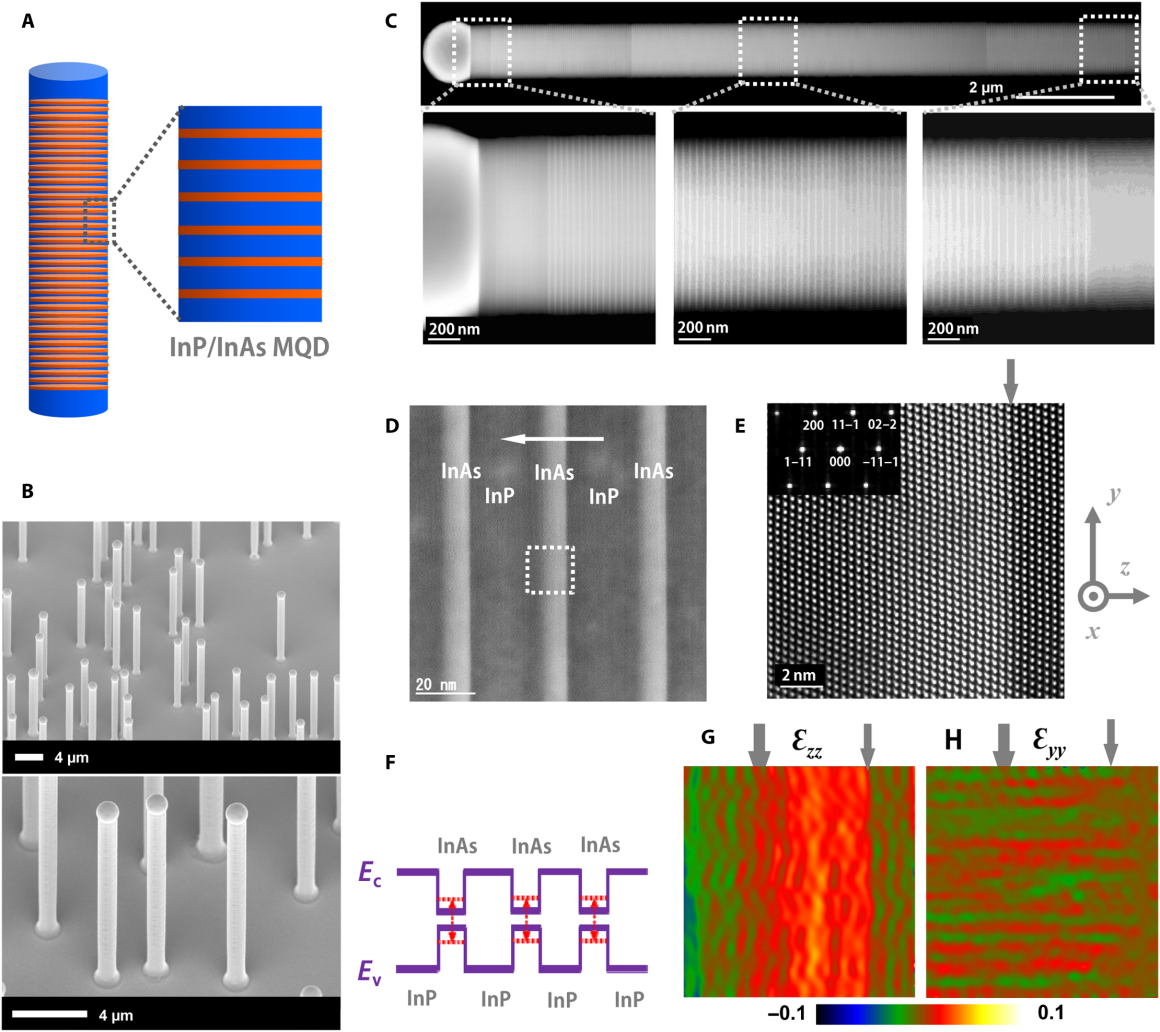
InP/InAs MQD nanowires. (**A**) Schematic diagram of MQD heterostructure nanowires and magnified view of the heterostructure highlighting the InP/InAs MQD structure. The InAs layer is indicated in red. (**B**) SEM images (tilt, 38°) of InP/InAs MQD nanowires grown on InP (111)B substrate. A particle can be seen at each nanowire tip. The nanowire diameters are 0.9 to 1.2 μm. The nanowire lengths are 9 to 12 μm. (**C**) HAADF-STEM images of an InP/InAs MQD nanowire taken along the [011] zone axis. The nanowire contains 400 units of InP/InAs heterostructure. (**D** and **E**) Aberration-corrected HAADF-STEM images of InP/InAs heterostructures taken along the [011] zone axis. The horizontal white arrow indicates the growth direction. The thickness of an InAs layer and an InP barrier layer are 9.0 ± 1 and 25.6 ± 1 nm, respectively. The square dotted region in (D) is shown in (E) with high resolution. The dislocation-free InAs/InP interface indicates a coherent growth despite a 3.1% lattice mismatch of InAs/InP. (**F**) Schematic band diagram of InP/InAs heterostructure nanowires with three InAs QDisks. The thickness as thin as ~9 nm of an InAs QDisk causes quantum confinement effect along the axial direction and thus increases the real gap energy compared with the bulk InAs gap energy (0.36 eV at 300 K), as indicated by red dotted lines. (**G** and **H**) Strain mapping of lattice spacing difference along the *y* and *z* directions for the STEM image shown in (E), indicating a compressive strain inside the InAs QDisk active region. The gray arrows indicate the InP/InAs interfaces.

To address the telecom-band single nanowire lasers, we have focused on indium-contained (InP and InAs) multi-quantum-disk (MQD) heterostructure (*33*, *34*) nanowires. We believe that the introduction of superlattice-like heterostructure into nanowires could offer advantages over homogeneous nanowire structures for designed laser wavelength output and in parallel with independent optimization of the cavity. Our MQD nanowire structure design consists of an InP nanowire, which functions as the primary part of the optical cavity by two facet mirrors, and an InP/InAs MQD, which serves as wavelength-tunable gain medium (Fig. 1A). An MQD structure was chosen because of its potential as a low-threshold and tunable gain medium for quantum confinement in a relatively wide wavelength range (*35*). In comparison with multi-quantum-well planar structures, which could produce dislocations due to the lattice mismatch as high as 3.1% InP/InAs, the nanowire heterostructure could endure large lattice mismatch (*36*) and thus has the advantage of producing a dislocation-free active region (*37*). High compressive strain can be introduced in InAs quantum disk (QDisk) by the high lattice mismatch (3.1%) of the InP/InAs material system (*38*). Theoretically and experimentally, it has been predicted and proven that strain could increase optical gain through strain engineering of band structure (*39*, *40*).

From the point view of the synthesis approach, most nanowires have been synthesized by selective area epitaxy (*16*, *19*, *23*, *28*, *29*). Because of its high controllability in terms of composition, heterostructure, doping, diameter, etc., the vapor-liquid-solid (VLS) mode has been widely used for the synthesis of nanowires and is considered to be a versatile and promising approach for next-generation building blocks. The Au-catalyzed VLS mode has been mostly used in the synthesis of various nanowires including GaAs and InP nanowires (*18*, *24*, *27*, *41*). However, the use of Au material is not compatible with the mainstream Si-based complementary metal-oxide semiconductor (CMOS) technology and thus hinders the integration of optically active III-V compound semiconductor nanowires into the CMOS process (*42*). We chose the self-catalyzed VLS approach in this work because of its high potential application in the field of Si-based integrated optoelectronics for the CMOS-compatible process feature (*42*), i.e., gold-free characteristics. Despite the use of gallium-catalyzed VLS approach for the InGaAs and GaAsSb nanowires, the wavelength of these nanowire lasers has not been taken into telecom-band range (*17*, *30*, *31*). Here, we chose the indium-contained InP/InAs heterostructure nanowires. On the one hand, the material combination enables the use of self-catalyzed, i.e., indium-catalyzed, VLS approach for the heterostructure formation along the axial direction. On the other hand, the InAs QDisk can function as wavelength-tunable gain medium covering a wide range, including the telecom band by quantum confinement effect along the axial direction.

## RESULTS

### Synthesis of InP/InAs heterostructure nanowires

We prepared MQD heterostructure nanowires by the metal organic vapor phase epitaxy (MOVPE) system, where the InP/InAs nanowire was grown via indium particle–catalyzed (or self-catalyzed) VLS mode. We used InP (111)B as the substrate for vertically aligned nanowires on the substrate by epitaxial growth, as shown in the scanning electron microscopy (SEM) images (Fig. 1B). High-angle annular dark-field scanning transmission electron microscopy (HAADF-STEM) data recorded with the electron beam along the [011] direction (Fig. 1C) directly reveal the MQD stacking along the growth direction. The nanowire is dominated by the growth via the VLS mode along the axial direction. There is negligible growth occurring on the side surface via the VPE mode because of the growth temperature as low as 350°C. The high growth controllability ensures the homogeneity of gain medium throughout the whole MQD region.

We have exploited high-resolution STEM imaging of the MQD nanowire structure to directly visualize the stacking of InP/InAs crystalline layers and the interface property (Fig. 1, D and E). Despite a 3.1% lattice mismatch between InP and InAs, the coherent growth results in a dislocation-free InAs/InP interface. The InP→InAs interface, as indicated by the gray arrow in Fig. 1E, is atomically abrupt, while the InAs→InP interface exhibits several-monolayer thickness due to the memory effect of As in indium catalyst particles. The corresponding electron diffraction pattern indexed for the [011] zone axis (the inset in Fig. 1E) indicates that both InAs QDisk and InP barrier layer have a zinc-blende crystalline structure without any stacking faults, which are usually present in VLS-grown III-V compound semiconductor nanowires. We found out that there are many stacking faults in an InP barrier layer, while very few stacking faults in InAs QDisks by analyzing over 20 QDisks. This might be attributed to the use of V/III ratio as high as 298, which might favorably suppress the defect formation of stacking faults. The high crystalline feature of InAs QDisks is the key point for superior optical property and hence may cause lasing at room temperature. The thickness as thin as ~9 nm, which is quite smaller than the Bohr radius (~34 nm) of bulk InAs, of an InAs QDisk causes quantum confinement along the axial direction (Fig. 1F), which enables much more gain attainable from InAs QDisks (*35*). The coherent growth of InP/InAs MQD structure indicates relatively high strain due to the lattice mismatch as high as 3.1%. We further carried out strain analysis for the STEM image shown in Fig. 1E by geometrical phase analysis (Fig. 1, G and H). Because a single InP barrier layer is quite thicker than a single InAs QDisk, high compressive strain is applied on each InAs QDisk. The lattice along the *y* direction remains coherent, while the lattice along the *z* direction is elongated in the InAs QDisk to relax strain energy. The compressively strained InAs QDisk is potentially expected to have several advantages, such as improved band imbalance and reduced threshold (*39*, *40*).

### Optical characterization of nanowire lasers

**Fig. 2:**
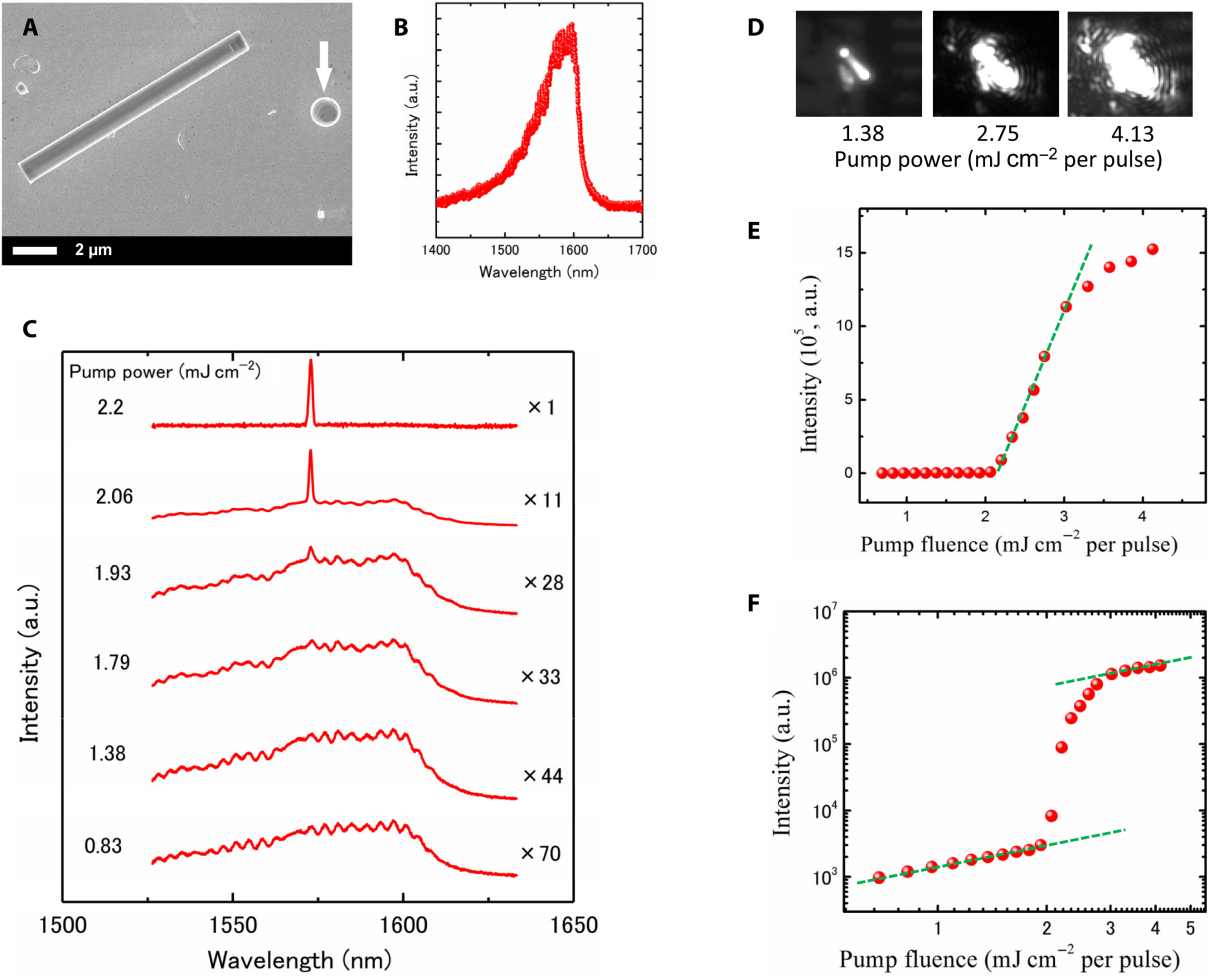
Optical characterization at room temperature. (**A**) SEM image of an InP/InAs MQD nanowire dispersed onto SiO_2_/Si substrates covered with a 200-nm-thick gold film for optical measurement. The white arrow indicates an indium particle moved from a nanowire tip in mechanical dispersion process. (**B**) PL spectrum of a single nanowire under a pump laser power of 0.83 mJ cm^−2^. (**C**) PL spectra of the nanowire with increasing pump power. The PL intensity continuously increases and eventually a narrow peak appears. Spectra are offset for clarity. (**D**) Far-field optical image of spatially resolved light emission from individual nanowires with increasing pump power. The diffraction fringes can be seen from the images with pump power of 4.13 mJ cm^−2^, indicating a lasing action. (**E**) L-L (light input-output) curve of the excitation power dependence. The curve reveals a transition from spontaneous emission to stimulated emission. The threshold power is estimated to be 2.15 mJ cm^−2^ per pulse. (**F**) L-L curve with semi logarithmic axis. Comparison of the measured output power data with a fit derived from the rate equations yields a spontaneous emission factor, β, of 0.0001 to 0.001. The S-shaped curve in the pump power dependence is indicative of the lasing action in the nanowire. a.u., arbitrary units.

We have characterized the optical properties of the dispersed InP/InAs MQD nanowires (Fig. 1A) using photoluminescence (PL). PL spectrum of individual nanowires reveals a relatively broad spontaneous emission with a dominant peak at 1.57 to 1.59 μm in telecom band (Fig. 2B). The result indicates that the energy of photon emission originated from InAs QDisks is quite enlarged compared with bulk InAs by quantum confinement along the axial direction. We further carried out excitation power–dependent measurement for the nanowire (Fig. 2C). As power density increased, the end emission intensity increased rapidly and became dominant. Above the excitation power of 2.15 mJ cm^−2^, the spontaneous peak collapsed into a narrow peak centered around 1573 nm. The full width at half maximum intensity is less than 1.2 nm.

Under the pump power higher than 2.15 mJ cm^−2^, far-field optical images show diffraction fringes (Fig. 2D), which originated from the diffraction of two coherent light beams coming out from the two end-facet mirrors. The L-L (light input-output) curve of the excitation power dependence reveals a transition from spontaneous emission to stimulated emission together with a narrowing line width (Fig. 2E). These features together with the observed characteristic nonlinear spectrally integrated output power versus pump power density (Fig. 2F) are clear indications of lasing behavior (figs. S1 and S2). Rate equation analysis was used to fit the experimental L-L curve and estimate the spontaneous emission factor β (section S2). The curve with β = 0.002 best fits the experimental data (fig. S3). Note that our nanowires were not capped by high-bandgap materials. Since InAs nanowires exhibit a surface recombination velocity as high as 3.0 × 10^3^ cm/s (*41*), the bare surface results in nonradiative recombination centers on the surface and, hence, considerably reduces excitation efficiency and β value. The surface effect on the excitation efficiency will be further discussed later.

To confirm the lasing mode, we calculated the mode spacing using Δλ = λ^2^/2*Ln*_e_, where λ is the emission wavelength, *n*_e_ is the effective refractive index, and *L* is the nanowire cavity length. For λ = 1603 nm, *n*_e_ = 3.0, and *L* = 10 μm, the predicted mode spacing is around 42.8 nm, which is comparable to the measured mode spacing of 32 nm (fig. S4). These results indicate that the lasing is the longitudinal mode in the nanowire Fabry-Pérot cavity structure.

**Fig. 3:**
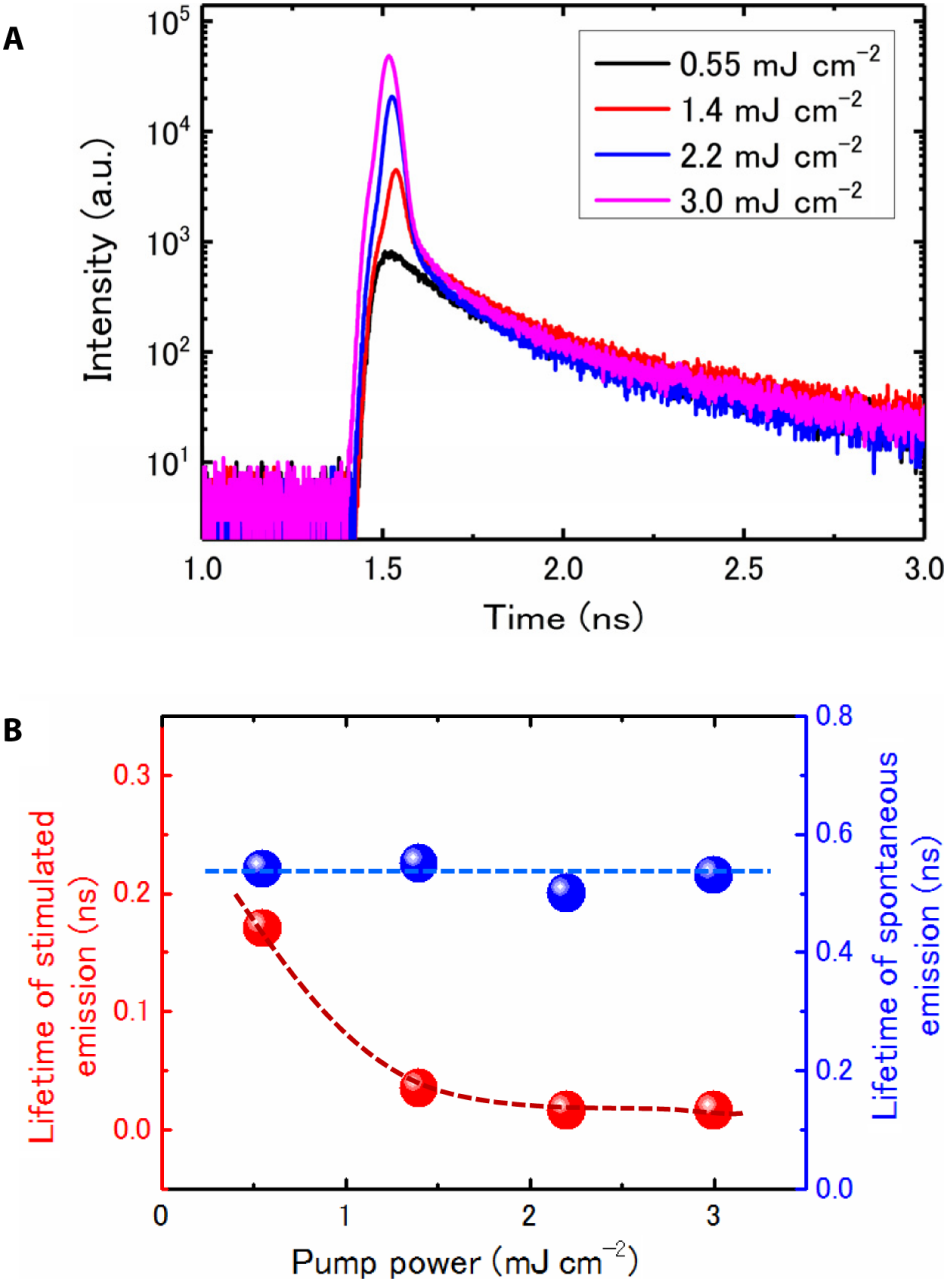
Time-resolved PL measurements using a single-photon detector with a time resolution of ~16 ps under increasing pump power at room temperature. (**A**) The PL decay curves with increasing pump power. For the power higher than 1.4 mJ cm^−2^, there are two decay times, namely, spontaneous and stimulated emission. (**B**) Lifetime of spontaneous and stimulated emission under varied pump power. The lifetime of spontaneous emission remains at the same level of 0.5 to 0.54 ns, while the stimulated emission shows faster lifetime with increasing pump power and eventually approaches a constant level of 0.016 ns, which is the resolution of the detector. The error bar is smaller than the dot size. The dashed lines are guides to the eyes. These features confirm the lasing behavior in the nanowire.

We have further carried out time-resolved PL measurement at room temperature to clarify the evolution of PL decay process under increasing pump power (Fig. 3) (*43*). Above the threshold, the decay time is limited by the resolution of the detector (fig. S5). The trends of the time-resolved PL data, although limited by the resolution, indicate a progressive shortening of the time constants with increasing pump power, indicating that the stimulation emission is becoming dominant (Fig. 3A). Note that Auger recombination may also affect the decay under high pump power because the Auger recombination rate is proportional to N^3^, where N is the carrier density (here, we suppose that the electron and hole concentration is equal for optical pump). We plotted the dependence of decay time on pump power (fig. S6A). With decreasing lifetime, the PL shows continuously increasing intensity. This indicates that the shortened lifetime is dominantly induced by stimulated emission. The decay evolution is a further indication of lasing behavior in the nanowire (*43*, *44*).

Above the lasing threshold, the lasing peak shows broadening line width and blue shift with increasing pump power (fig. S6B). The broadening peak above the threshold might be induced by temperature increase of the nanowire due to the heating effect by intense laser irradiation. The tiny blue shift (~1 nm) of the lasing peak might be due to the change of effective refractive index resulting from the high carrier concentration under intense laser irradiation. One more issue might be the blue shift of effective bandgap originated from quantum-confined stark effect due to screening effect by high carrier concentration, since the <111>-oriented MQD InP/InAs nanowire has strong spontaneous and piezoelectric polarizations (*45*).

About the stability of nanowire lasing, we first conducted the lasing measurement at cryogenic temperatures (liquid He, 4 K). The lasing state was very stable and reproducible. This is attributed to the cryogenic temperature and vacuum environment. The heating effect induced by lasing pump could be effectively suppressed. We then performed the measurement at room temperature, and the surrounding atmosphere of nanowire samples was air. So the heat effect by laser irradiation may be serious and probably cause burning out of nanowires. However, later, we found that using the substrate with high thermal conductivity of a gold film was very helpful to suppress the heating effect. That is why we used gold film–covered SiO_2_/Si substrate for nanowire dispersion. Figure S7 shows lasing spectra recorded at different dates (1-week difference) for a same nanowire. The nanowire shows reproducible lasing behavior even 1 week after the initial lasing measurement. In general, most nanowires showed stable lasing operation despite the surrounding air atmosphere and room temperature conditions.

### Tuning lasing wavelength range

**Fig. 4:**
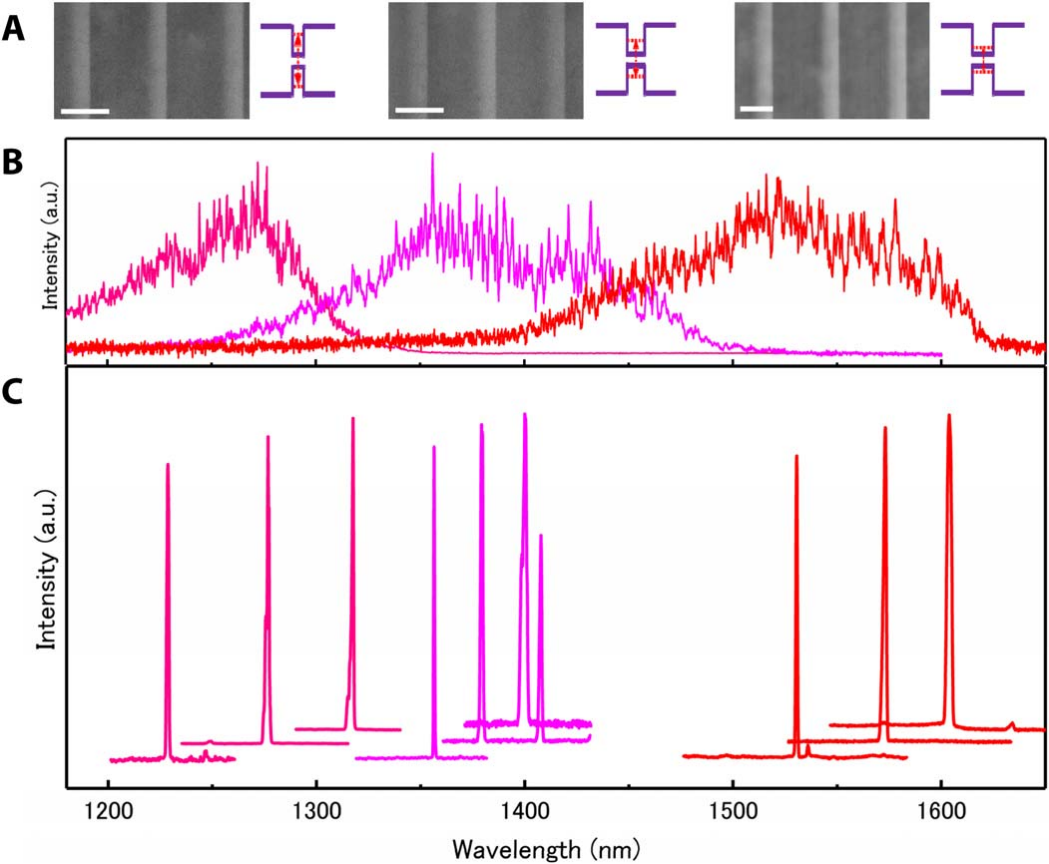
Tuning the laser wavelength range in telecom band. (**A**) Aberration-corrected HAADF-STEM images of InP/InAs MQD nanowires taken along the [011] zone axis. These nanowires were grown with increased flow rates of TMIn:TBA (μmol/min): 0.75:223, 0.98:290, and 1.5:447 from the left-hand side to right-hand side. The flow rates of TMIn:TBA modulate the thickness of the InAs QDisk. From the left-hand side to right-hand side, the thickness of a single InAs QDisk is 6.8 ± 0.8, 7.5 ± 0.8, 9.0 ± 1 nm, respectively. Scale bars, 20 nm. The insets schematically indicate how the real gap energy (ground state) of the InAs QDisk is modulated by the thickness through quantum confinement along the axial direction. (**B**) Corresponding spontaneous PL spectra of individual nanowires shown in Fig. 4A. The dominant PL peak is shifted up to the high-energy, i.e., short-wavelength, side with decreased flow rates. This indicates enhanced quantum confinement by reduced thickness of a single InAs QDisk. (**C**) Corresponding PL spectra of stimulated emission. Spectra are offset for clarity. The laser wavelength range is modulated by the growth parameters, i.e., flow rates of TMIn and TBA sources. Thus, a broad wavelength range is covered in the whole telecom band, including two technologically important telecom-band windows of 1.3 and 1.55 μm.

The tunability of laser wavelength in a broad range, e.g., the full telecom band (1.2 to 1.6 μm), is considerably important for the development of practical applications. The MQD heterostructure serving as the active region provides the ability to modulate the photon emission energy by tuning the thickness of the InAs QDisk in a broad range through quantum confinement (Fig. 4A). The thickness of the InAs QDisk usually can be tuned by parameters of growth time and flow rates of source materials. We found out that the growth time of 0.3 s for MQD nanowires with PL peak centered at 1.57 to 1.59 μm is the minimum time, which is limited by the MOVPE system. Hence, this makes it difficult to make thinner InAs QDisks by growth time. We then found that we could tune the thickness for thinner InAs QDisks by the parameter of flow rate of source materials (Fig. 4A). The STEM images indicate that the thickness of InAs QDisks is reduced with decreased flow rates of trimethylindium (TMIn) and tertiarybutylarsenic (TBA).

The spontaneous emission PL spectra show that the dominant peak is shifted up to the high-energy (short-wavelength) side with reducing thickness of single InAs QDisks (Fig. 4B), indicating that the modulation of the photon emission range is driven by quantum confinement effect along the axial direction. On the basis of the thickness of single InAs QDisks, we calculated the bandgap energy (ground state) of InAs QDisks without considering strain (table S2). There is a deviation between calculated bandgap energy and experimental PL peak range, which may be attributed to the high compressive strain in the InAs QDisks in MQD heterostructure nanowires (Fig. 1). The compressive strain changes the band structure together with the enlargement of bandgap energy. The result is in agreement with the strain analysis in MQD heterostructure nanowires (Fig. 1).

We have then observed the stimulation emission for a number of nanowires by increasing excitation power (Fig. 4C). For nanowires grown under specific flow rates, there is a variation of laser wavelength mainly resulting from the varied cavity (nanowire) length due to the mechanical dispersion of nanowires. The laser wavelength of nanowires grown under each flow rate (different colors in Fig. 4C) is located in different ranges and increases with thickness of InAs QDisks. The broad wavelength range covers the full telecom band, including two extremely important telecom-band windows of 1.3 and 1.55 μm. These results indicate a high capability of MQD heterostructure nanowires to modulate the lasing wavelength in a broad range by the thickness of each InAs QDisk.

## DISCUSSION

Low-threshold nanowire lasers are technologically preferred for potential practical application in optoelectronics. The threshold of our nanowire lasers is in the range of 1.4 to 4.2 mJ cm^−2^ per pulse. The value is about 10 times higher than those of GaAs nanowires (~0.2 mJ cm^−2^ per pulse) (*18*) and InGaAs quantum dot nanowires (~0.2 mJ cm^−2^ per pulse) (*19*). We consider the possible reason below, which might be responsible for the high threshold. Our nanowires are not capped by high-bandgap materials, as we found that growth of a shell layer results in a tapering tip and thus breaks the FP cavity structure. Since InAs nanowires exhibit a surface recombination velocity as high as 3.0 × 10^3^ cm/s (*41*), the bare surface results in nonradiative recombination centers on the surface and hence considerably reduces excitation efficiency. Treu *et al.* (*46*) have reported that up to ~100 times enhancements of the emission intensities from InAs nanowires could be obtained by growing an InAsP shell to produce core-shell structure and thus suppress nonradiative recombination formation. This might be the most important reason for the low threshold because of the remarkable effect of a capping layer on improving excitation efficiency (*17*–*19*, *23*). We believe that low threshold and/or further continuous wave lasing may be expected by optimizing the MQD heterostructure, e.g., growing a capping layer to passivate the surface while keeping the cavity structure and reducing the number of InAs QDisks to avoid the absorption loss.

Despite the high surface recombination velocity of the InAs QDisk active region, our telecom-band single nanowire lasers with a bare surface still operate at room temperature. This indicates that its superior optical property presumably originated from the high-quality MQD heterostructure and high strain in InAs QDisk active region. The strain has been theoretically and experimentally found to be very favorable on improving quantum well laser performance (*39*, *40*); for instance, (i) increasing laser gain by improving band imbalance and consequently reducing threshold pump power, and (ii) the reduced threshold pump power decreases the Auger recombination loss, which becomes serious with longer wavelength. We consider that strain may be quite favorable for lasing operation in our highly strained MQD heterostructure nanowires. We analyze the strain situation and discuss the strain effect on nanowire lasing below.

Because of the relatively large diameter (~1 μm), we consider our MQD InP/InAs nanowire as strained quantum wells with a vertical wire structure. The InP/InAs heterostructure interface exhibits coherent growth despite as high as 3.1% lattice mismatch. We calculated the strain in our MQD InP/InAs nanowires (see Supplementary Text)

$$\varepsilon_{xx}=\varepsilon_{yy}=\varepsilon_{zz}=-1.5\%,\varepsilon_{xy}=\varepsilon_{yz}=\varepsilon_{zx}=1.6\%$$

This indicates a compressive strain in the growth (111) plane and a tensile strain along the perpendicular <111> direction. This high strain has also been verified by transverse optical phonon shift to the high energy side as large as 15 cm^−1^ in micro-Raman measurement (*38*). The axial strain breaks the cubic symmetry of the InAs semiconductor active layer. This splits the degeneracy of the light- and heavy-hole states at Г, typically about 60 meV for ε_∥_ ≅ 1 %, and introduces an anisotropic valence band structure. The splitting energy can be further enlarged by quantum confinement effect and can be far higher than the thermal energy, *k*_*B*_T, at room temperature. Thus, such high strain can highly benefit gain increase and consequently reduce threshold pump power (*39*, *47*, *48*). The Auger recombination process, one of the main nonradiative loss mechanisms, which increases with longer wavelength, can also be considerably suppressed by reduced threshold pump power.

## CONCLUSION

We demonstrate optically pumped telecom-band single nanowire lasers operating at room temperature based on MQD InP/InAs heterostructure nanowires. Our work represents a new level of complexity in nanowire structures with working wavelength in telecom-band range by a gold-free CMOS-compatible synthesis approach (*49*). The demonstration of telecom-band nanowire lasers operating at room temperature is a major step forward in providing practical integrable light sources for optoelectronics and data communication.

## MATERIALS AND METHODS

### Nanowire synthesis

We synthesized the MQD InP/InAs heterostructure nanowires in a MOVPE system. Indium particles were formed on InP substrate by introducing TMIn source material for 15 min at a flow rate of 3.0 μmol/min and temperature of 360°C, as we have shown before (*38*, *50*). The temperature was then reduced to the growth temperature (350°C), and growth was initiated by introducing TMIn and TBA or tertiarybutylphosphine (TBP) simultaneously. For the InP segment growth, the flow rates of TMIn and TBP were 2.12 to 3.03 μmol/min and 803.6 μmol/min, respectively. For MQD nanowires, the growth times for the InP and InAs segments were 10 and 0.3 s, respectively.

### Electron microscopy measurement

The morphology of the nanowires was analyzed using a scanning electron microscope (Ultra 55, Carl Zeiss) at an accelerating voltage of 5 to 15 kV. The cross-sectional samples used for the TEM measurements were prepared by using a focused ion beam (operated at 30 kV; SII SMI-3050SE) system. The STEM analysis was performed in a JEM2100F (operated at 200 kV; JEOL) equipped with a HAADF detector and an x-ray energy-dispersive spectrometer. The aberration-corrected STEM analysis was carried out in a JEM-ARM200F (operated at 200 kV; JEOL) equipped with a HAADF detector. The strain analysis of MQD nanowires was performed by GPA software (HREM Research Inc., www.hremresearch.com/).

### Optical measurement

We mechanically dispersed nanowires onto a gold film–covered SiO_2_/Si substrate. The indium particles at the nanowire tips could be moved from the nanowire in the mechanical dispersion process. Hence, two (111) facet mirrors could be resulted at both ends of a nanowire and hence construct a Fabry-Pérot cavity because of the refractive index difference between the nanowire and the surrounding air.

A mode-locked Ti:sapphire laser delivering several-picosecond pulses was used to pump a single nanowire. The pump wavelength was fixed at 800 nm with a repetition rate of 80 MHz. The pump laser spot with a circular shape was focused down to 2.0-μm diameter using 50× lens with a numerical aperture of 0.42. The detection parts contain a visible near-infrared CCD (charge-coupled device) camera and a highly sensitive short-wave infrared InGaAs photodiode array CCD with a long-wave-pass filter (cutoff wavelength of 1100 nm) for the microscope image and the PL image, respectively. To measure the PL spectrum, we coupled the emission into the multimode fiber and directed it to a grating spectrometer with a cooled InGaAs array.

With regard to the time-resolved PL measurement, we used a femtosecond Ti:sapphire laser (repetition rate of 80 MHz) as a pump laser, a superconducting nanowire single-photon detector, and a time-correlated single-photon counting module for our time-resolved PL (*43*). We measured the half width at half maximum of the detected pump pulse to be 16 ps (fig. S4), which directly corresponds to the minimum decay rate of the emission lifetime we can measure.

## Supplementary Material

http://advances.sciencemag.org/cgi/content/full/5/2/eaat8896/DC1

## SUPPLEMENTARY MATERIALS

Supplementary material for this article is available at http://advances.sciencemag.org/cgi/content/full/5/2/eaat8896/DC1 Supplementary Text Section S1. Calculation of electric field dispersion relationships Section S2. Rate equation analysis Section S3. Strain analysis Fig. S1. Calculation of electric field dispersion relationships for single InP nanowires dispersed on Au/SiO_2_/Si substrate at the wavelength of 1550 nm. Fig. S2. Calculation of electric field dispersion relationships for single InP nanowires dispersed on SiO_2_/Si substrate at the wavelength of 1550 nm. Fig. S3. Rate equation analysis of experimental data. Fig. S4. PL spectra of a nanowire under stimulated emission. Fig. S5. Time-resolved decay of nanowire lasing and system function. Fig. S6. Delay, lifetime, lasing peak line width and shift measured as a function of pumping power. Fig. S7. Lasing spectra recorded at different period (1 week) for a same nanowire. Table S1. Parameters used in rate equation analysis. Table S2. Thickness of a single InAs QDisk versus calculated bandgap energy (without strain) and PL peak range of spontaneous emission (compressively-strain in MQD InP/InAs heterostructure nanowires) at room temperature.
