# Supplementary material for: Telecom-band lasing in single InP/InAs heterostructure nanowires at room temperature
Source: Sci Adv. 2019 Feb 22;5(2):eaat8896. doi: 10.1126/sciadv.aat8896 (PMC6386577; doi:10.1126/sciadv.aat8896)
Supplement: http://advances.sciencemag.org/cgi/content/full/5/2/eaat8896/DC1 [file supp_5_2_eaat8896__index.html]

Science Advances | Science Advances

## Supplementary Materials

**This PDF file includes:**

- Supplementary Text
- Section S1. Calculation of electric field dispersion relationships
- Section S2. Rate equation analysis
- Section S3. Strain analysis
- Fig. S1. Calculation of electric field dispersion relationships for single InP nanowires dispersed on Au/SiO2/Si substrate at the wavelength of 1550 nm.
- Fig. S2. Calculation of electric field dispersion relationships for single InP nanowires dispersed on SiO2/Si substrate at the wavelength of 1550 nm.
- Fig. S3. Rate equation analysis of experimental data.
- Fig. S4. PL spectra of a nanowire under stimulated emission.
- Fig. S5. Time-resolved decay of nanowire lasing and system function.
- Fig. S6. Delay, lifetime, lasing peak line width and shift measured as a function of pumping power.
- Fig. S7. Lasing spectra recorded at different period (1 week) for a same nanowire.
- Table S1. Parameters used in rate equation analysis.
- Table S2. Thickness of a single InAs QDisk versus calculated bandgap energy (without strain) and PL peak range of spontaneous emission (compressively-strain in MQD InP/InAs heterostructure nanowires) at room temperature.

Download PDF

**Files in this Data Supplement:**

- Adobe PDF - aat8896\_SM.pdf
