# Supplementary material for: Telecom-band lasing in single InP/InAs heterostructure nanowires at room temperature
Source: Sci Adv. 2019 Feb 22;5(2):eaat8896. doi: 10.1126/sciadv.aat8896 (PMC6386577; doi:10.1126/sciadv.aat8896)
Supplement: http://advances.sciencemag.org/cgi/content/full/5/2/eaat8896/DC1 [file aat8896_SM.pdf]

Science Advances
AAAS

advances.sciencemag.org/cgi/content/full/5/2/eaat8896/DC1

# Supplementary Materials for

## Telecom-band lasing in single InP/InAs heterostructure nanowires at room temperature

Guoqiang Zhang*, Masato Takiguchi, Kouta Tateno, Takehiko Tawara, Masaya Notomi, Hideki Gotoh

*Corresponding author. Email: guoqiang.zhang.ta@hco.ntt.co.jp

Published 22 February 2019, *Sci. Adv.* **5**, eaat8896 (2019)
DOI: 10.1126/sciadv.aat8896

**This PDF file includes:**

Supplementary Text
Section S1. Calculation of electric field dispersion relationships
Section S2. Rate equation analysis
Section S3. Strain analysis
Fig. S1. Calculation of electric field dispersion relationships for single InP nanowires dispersed on Au/$SiO_2$/Si substrate at the wavelength of 1550 nm.
Fig. S2. Calculation of electric field dispersion relationships for single InP nanowires dispersed on $SiO_2$/Si substrate at the wavelength of 1550 nm.
Fig. S3. Rate equation analysis of experimental data.
Fig. S4. PL spectra of a nanowire under stimulated emission.
Fig. S5. Time-resolved decay of nanowire lasing and system function.
Fig. S6. Delay, lifetime, lasing peak line width and shift measured as a function of pumping power.
Fig. S7. Lasing spectra recorded at different period (1 week) for a same nanowire.
Table S1. Parameters used in rate equation analysis.
Table S2. Thickness of a single InAs QDisk versus calculated bandgap energy (without strain) and PL peak range of spontaneous emission (compressively-strain in MQD InP/InAs heterostructure nanowires) at room temperature.

**Supplementary Text**

**Section S1. Calculation of electric field dispersion relationships**

In our optical characterization experiment, MQD nanowires were dispersed onto $SiO_2$/Si substrates covered with a 200-nm-thickness gold film. The gold film is favorable to suppress the heating effect induced by optical pumping. But the gold film may also cause the plasmonic lasing mode. To clarify the lasing mode, we carried out the following calculation.

Figures S1 and S2 compare the theoretical electric field dispersion relationships for the nanowires on gold and $SiO_2$ substrates. Finite Element Method simulation (Comsol) was used to model the behavior of the simulated nanowires. A wavelength of 1550 nm was used to model an electric field in a 450-nm-diameter nanowire. In fig. S1a and fig. S2a, effective mode index is compared to the diameter of the nanowire. Lines 1 to 16 are electric modes which can exist in the InP nanowire. Modes 1 and 2 are fundamental modes, normally called $HE_{11.}$ They usually only degenerate in symmetrical systems (such as in optical fibers) even though they have different polarizations. In this case, for the nanowires dispersed on the gold surface, mode degeneration disappears, and the nanowire begins to exhibit plasmonic-like modes (hybrid mode) (fig. S1b). Here, it is shown that plasmonic modes can appear when the diameter is smaller than about 600 nm. The electric modes which were localized in our nanowires were not plasmonic modes, where the electric field magnitude is concentrated near the gold surface, but photonic modes, as the diameter of the nanowire was too thick to support the plasmonic mode. It is found that the mode index is ~3 (fig. S1d) obtained from the free spectrum range, further suggesting that the electrical mode is fundamental and photonic.

We further carried out optical characterization by using $SiO_2$/Si substrates. We

did see lasing from multiple nanowires even using the substrate without gold film, one of which is shown in fig. S4. This is also a direct indicative of photonic mode.

**Section S2. Rate equation analysis**

Rate equation analysis was used to fit the experimental L-L curve (Fig. 2F) and estimate the spontaneous emission factor, β. The rate equation for the carrier density in the active region, N, and photon density in the cavity mode, S, under optical pumping are as follows

$$\frac{dN}{dt} = R_p - v_g G S - \frac{N}{\tau_r} - \frac{N}{\tau_{nr}}$$

$$\frac{dS}{dt} = v_g \Gamma G S - \frac{\beta \Gamma N}{\tau_r} - \frac{S}{\tau_p}$$

$$G = g(N - N_{tr})$$

The rate equations were numerically evaluated using the parameters shown in table S1. The curve with β = 0.002 best fits the experimental data (fig. S3).

**Table S1. Parameters used in rate equation analysis.**

| | | |
|---|---|---|
| g | differential gain coefficient | $8.8\times10^{-16}$ $cm^2$ * |
| $N_{tr}$ | carrier density for transparency | $1\times10^{16}$ $cm^{-3}$ * |
| $\tau_r$ | radiative recombination lifetime | 1.4 ns * |
| $\tau_{nr}$ | nonradiative recombination lifetime | 0.55 ns |
| $\tau_p$ | photon lifetime in cavity (Q=300@1.55um) | 1.55 ps |
| Γ | confinement factor | 0.04 |
| $V_g$ | group velocity | $1.2\times10^{10}$ cm/s |
| $V_m$ | mode volume (total volume of a nanowire) | $7.85\times10^{-8}$ $cm^3$ |

* values adopted from A. A. Ukhanov, A. Stintz, P. G. Eliseev, K. J. Malloy, Comparison of the carrier induced refractive index, gain, and linewidth enhancement factor in quantum dot and quantum well lasers. *Appl. Phys. Lett.* **84**, 1058-1060 (2004).

**The nanowire diameter is 1.2 µm. The nanowire length is 10µm. The active layer region includes 50 layers of InAs quantum disks. Thickness of each active layer is 10 nm.

**Section S3. Strain analysis**

We analyze the strain situation in our nanowires and discuss the effect on lasing operation below. Because of relatively large diameter (~ 1µm), we consider our MQD InP/InAs nanowire as strained quantum wells with a vertical wire structure. The InP/InAs heterostructure interface exhibits coherent growth despite as high as 3.1% ($\varepsilon_{\parallel} = -3.1\%$) lattice mismatch, indicating a compressive strain in the growth plane and tensile strain along the growth direction in InAs QDisks. In the case of strain layer grown on <111>

direction (for *fcc* lattice structure)

$$\sigma = \frac{C_{11} + 2C_{12} + 4C_{44}}{2C_{11} + 4C_{12} - 4C_{44}}$$

$$\varepsilon_{xx} = \left[\frac{2}{3} - \frac{1}{3}\left(\frac{2C_{11} + 4C_{12} - 4C_{44}}{C_{11} + 2C_{12} + 4C_{44}}\right)\right] \varepsilon_{\parallel}$$

$$\varepsilon_{yy} = \varepsilon_{xx}$$

$$\varepsilon_{zz} = \varepsilon_{xx}$$

$$\varepsilon_{xy} = \left[-\frac{1}{3} - \frac{1}{3}\left(\frac{2C_{11} + 4C_{12} - 4C_{44}}{C_{11} + 2C_{12} + 4C_{44}}\right)\right] \varepsilon_{\parallel}$$

$$\varepsilon_{yz} = \varepsilon_{xy}$$

$$\varepsilon_{zx} = \varepsilon_{yz}$$

where σ is known as Poisson's ratio and $C_{11}, C_{12}$ and $C_{44}$ are the elastic stiffness constants. For InAs semiconductors, $C_{12}$= 4.54 $\times 10^{11}$ dyn/cm$^2$, $C_{11}$= 8.34 $\times 10^{11}$ dyn/cm$^2$, and $C_{44}$= 3.95 $\times 10^{11}$ dyn/cm$^2$. Then the strain distribution can be obtained

$$\varepsilon_{xx} = \varepsilon_{yy} = \varepsilon_{zz} = -1.5\%$$

$$\varepsilon_{xy} = \varepsilon_{yz} = \varepsilon_{zx} = 1.6\%$$

The result indicates a compressive strain in the growth (111) plane and a tensile strain along the perpendicular <111> direction. The axial strain breaks the cubic symmetry of InAs semiconductor active layer. This splits the degeneracy of the light- and heavy-hole states at Γ and introduces an anisotropic valence band structure (*38, 39*).

Such coherent growth of InAs/InP with 3.1% lattice mismatch is extremely difficult to realize for conventional epitaxial growth with a film structure due to the limitation of critical thickness. Thus the effect of such high strain on quantum well band structure has not been well understood before, both theoretically and experimentally. The nanowire structure enables the growth of mismatch-dislocation-free materials with coherent interface because of its high capability to endure high lattice mismatch. From this point of view, to explore the effect of such high strain on band structure of highly-mismatched heterostructure nanowires may open up new opportunities to engineering band structures and developing new-principle devices.

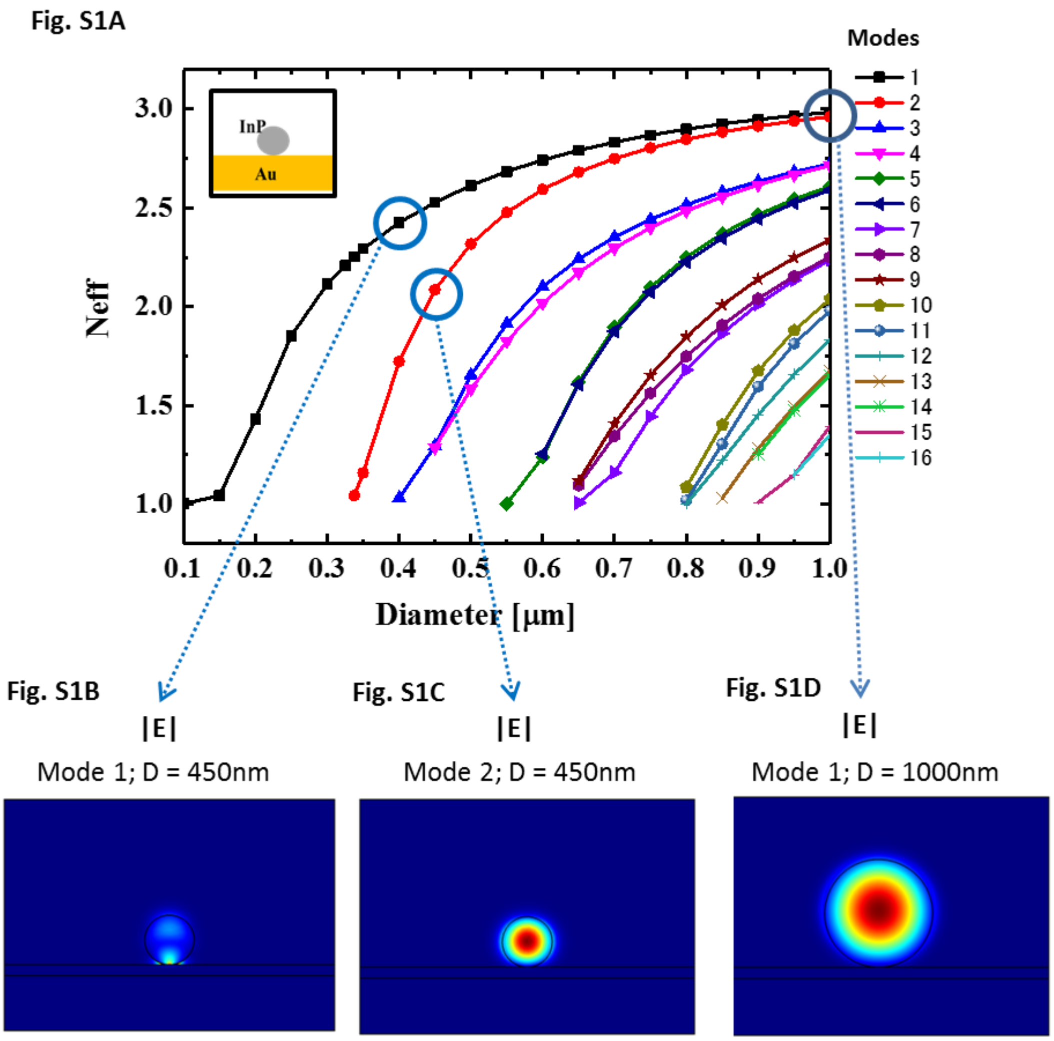


**Fig. S1. Calculation of electric field dispersion relationships for single InP nanowires dispersed on Au/$SiO_2$/Si substrate at the wavelength of 1550 nm.** The inset in fig. S1A is a schematic diagram of the sample structure.

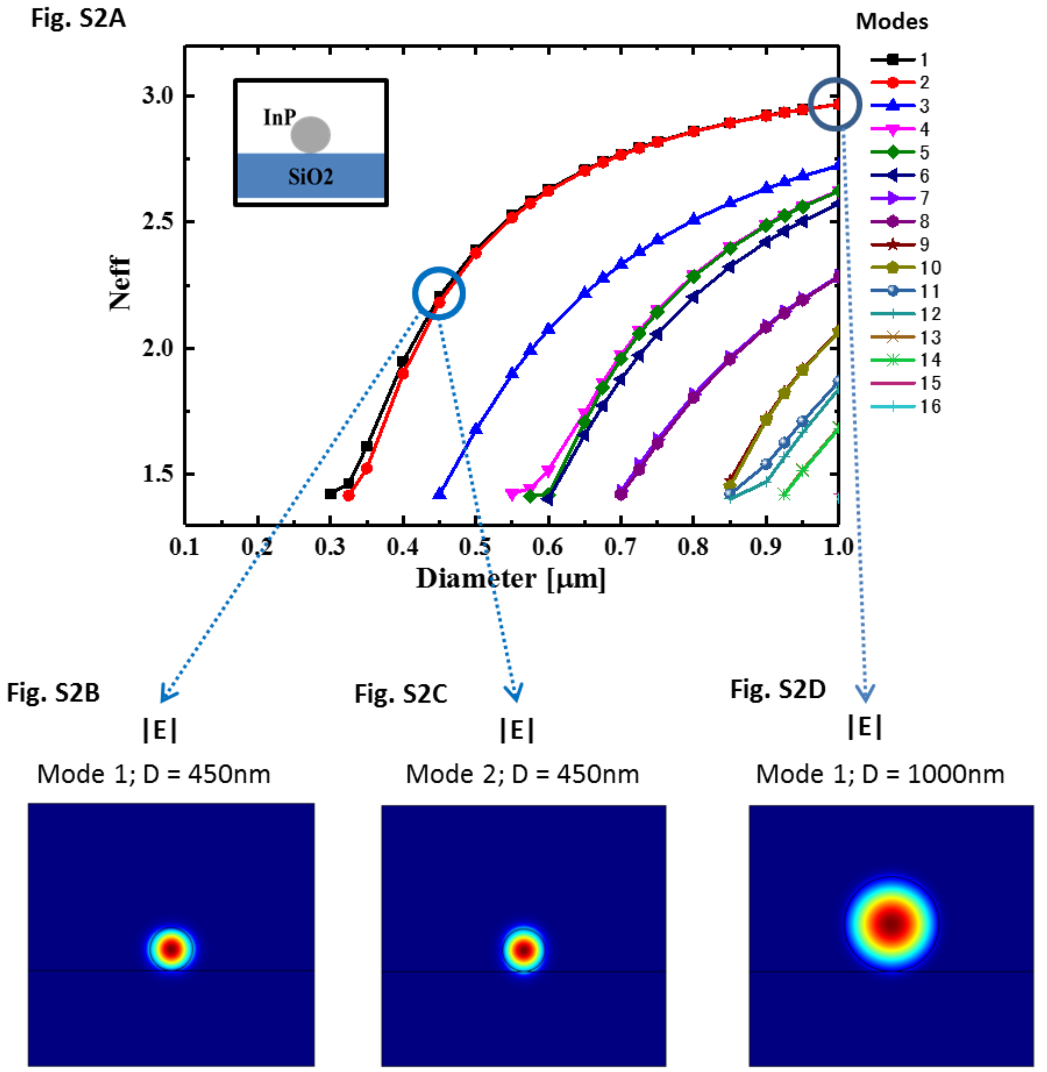


**Fig. S2. Calculation of electric field dispersion relationships for single InP nanowires dispersed on $SiO_2$/Si substrate at the wavelength of 1550 nm.** The inset in fig. S2A is a schematic diagram of the sample structure.

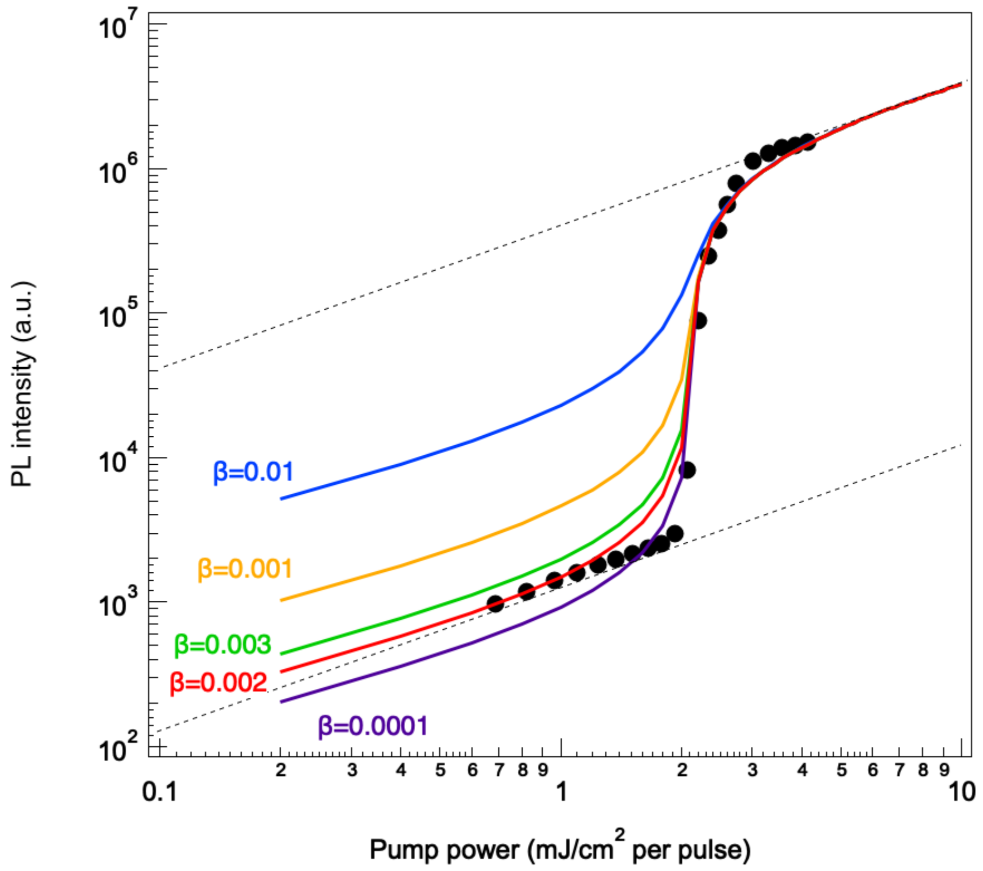


**Fig. S3. Rate equation analysis of experimental data.** Rate equation calculations for various values of spontaneous emission factor β. The curve with β = 0.002 best fits the experimental data (black dots).

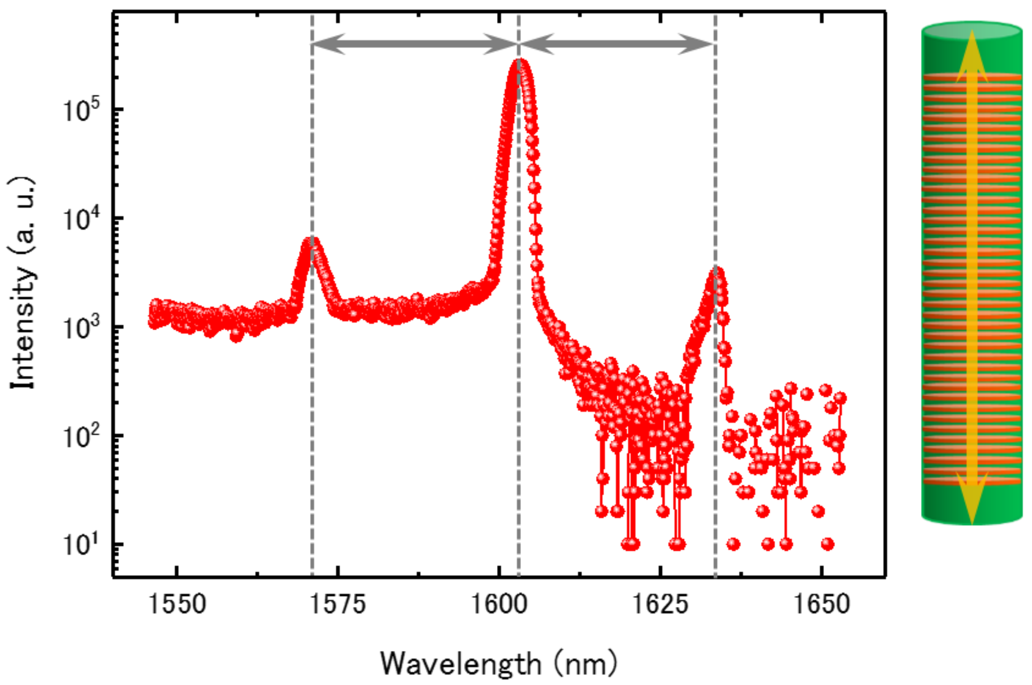


**Fig. S4. PL spectra of a nanowire under stimulated emission.** There are multiple peaks with the same distance of 32 nm. The inset shows a schematic diagram of the Fabry-Perot cavity structure in a nanowire.

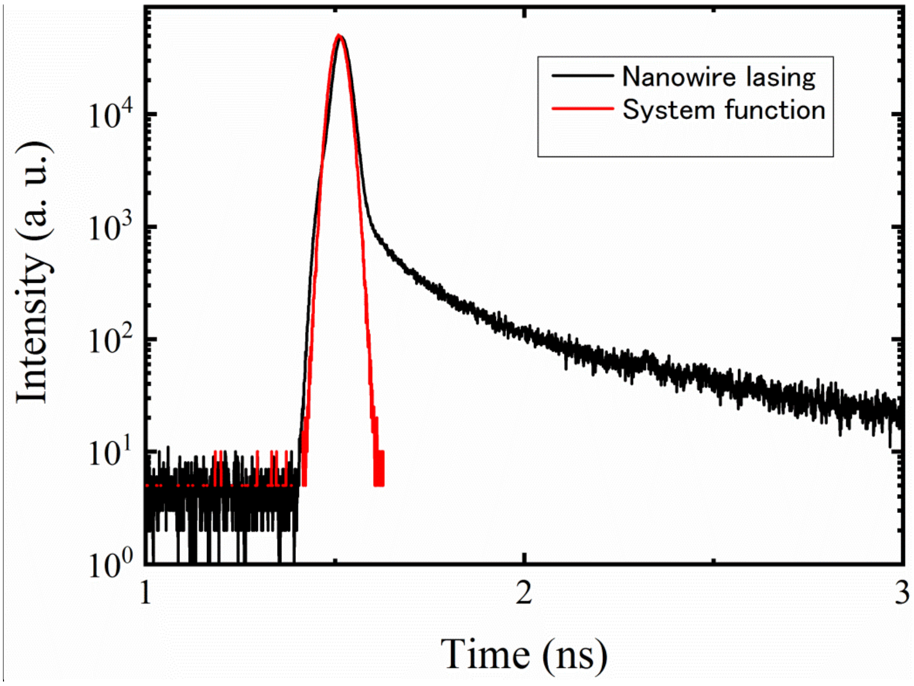


**Fig. S5. Time-resolved decay of nanowire lasing and system function.** We measured the half-width at half-maximum of the detected pump pulse to be 16 ps, which directly corresponds to the minimum decay rate of the emission lifetime we can measure.

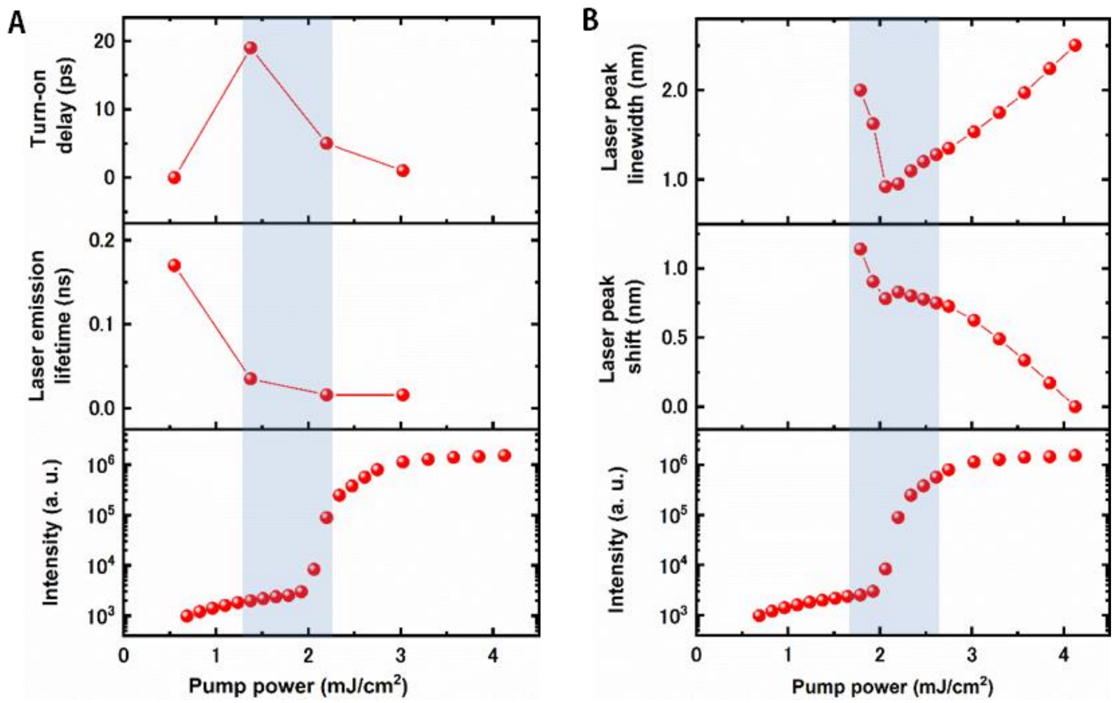


**Fig. S6. Delay, lifetime, lasing peak line width and shift measured as a function of pumping power.** (**A**) Turn-on delay and lifetime measured as a function of pumping power. The turn-on delay time with increasing pump power initially increases and then decreases after the nanowire starts lasing. This is a typical feature of lasing behavior. (**B**) Laser peak linewidth and shift measured as a function of pumping power.

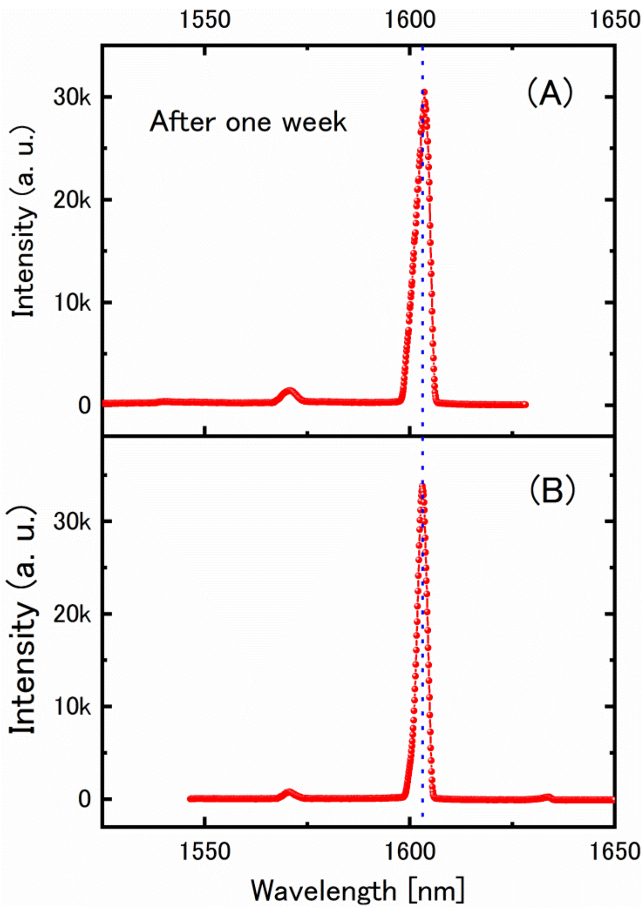


**Fig. S7. Lasing spectra recorded at different period (1 week) for a same nanowire.** (**A**) Lasing spectrum of the nanowire one week after the first time measurement. (**B**) Lasing spectrum in the first time measurement for the same nanowire after dispersion onto Au-covered $SiO_2$/Si substrate. Both measurement were conducted in air atmosphere. The vertical dotted line schematically shows the peak position, indicating a same peak wavelength.

**Table S2. Thickness of a single InAs QDisk versus calculated bandgap energy (without strain) and PL peak range of spontaneous emission (compressively-strain in MQD InP/InAs heterostructure nanowires) at room temperature.**

| **Thickness of a single InAs QDisk in MQD InP/InAs heterostructure nanowires (nm)** | **6.8±0.8** | **7.5±0.8** | **9.0±1** |
|---|---|---|---|
| Calculated band-gap energy (ground state) for InAs QDisks without strain (μm) | 1.66±0.2 | 1.83±0.2 | 2.14±0.2 |
| PL peak range of spontaneous emission for compressively-strained InAs QDisks in MQD InP/InAs heterostructure nanowires (μm) | 1.26±0.05 | 1.38±0.05 | 1.57±0.05 |
